# Safety assessments and clinical features of PARP inhibitors from real-world data of Japanese patients with ovarian cancer

**DOI:** 10.1038/s41598-024-63600-z

**Published:** 2024-06-01

**Authors:** Ryosuke Uekusa, Akira Yokoi, Eri Watanabe, Kosuke Yoshida, Masato Yoshihara, Satoshi Tamauchi, Yusuke Shimizu, Yoshiki Ikeda, Nobuhisa Yoshikawa, Kaoru Niimi, Shiro Suzuki, Hiroaki Kajiyama

**Affiliations:** 1 Department of Obstetrics and Gynecology, Nagoya University Graduate School of Medicine, Tsuruma-cho 65, Showa-ku, Nagoya, 466-8550 Japan; 2 Institute for Advanced Research, Nagoya University, Furo-cho, Chikusa-ku, Nagoya, 464-9601 Japan; 3 Department of Gynecologic Oncology, Aichi Cancer Center Hospital, Kanokoden 1-1, Chikusa-ku, Nagoya, 464-8681 Japan

**Keywords:** Ovarian cancer, Medical research

## Abstract

Poly (ADP-ribose) polymerase inhibitors have been increasingly used in ovarian cancer treatment. However, the real-world safety data of these drugs in Japanese patients are limited. This retrospective study included 181 patients with ovarian cancer who received olaparib or niraparib at two independent hospitals in Japan between May 2018 and December 2022. Clinical information and blood sampling data were collected. Regarding patient backgrounds, the olaparib group had higher proportions of patients with serous carcinoma, *BRCA* positivity, homologous recombination deficiency, and those receiving maintenance therapy after recurrence treatment than the niraparib group. Regarding toxicity properties, the most common reasons for discontinuation in the olaparib group were anemia, fatigue, and nausea, while the reason in the niraparib was thrombocytopenia. Thrombocytopenia caused by niraparib treatment occurred earlier than anemia caused by olaparib treatment. Patients with a low body mass index or who had undergone several previous treatment regimens were more likely to discontinue treatment within the first 3 months. Although we analyzed blood collection data, predicting treatment interruptions due to blood toxicity was challenging. In this study, we revealed the characteristics of patients and the timing of interruptions for each drug, highlighting the importance of carefully managing adverse effects.

## Introduction

Ovarian cancer is the third most common gynecologic malignancy and was the second leading cause of death from gynecologic cancer worldwide in 2020^1^. Epithelial ovarian cancer constitutes most ovarian malignancies, with most cases diagnosed at an advanced stage. The 5 years survival rate for ovarian cancer is approximately 30%. Despite achieving an approximately 80% response rate with standard treatment of optimal debulking surgery and platinum-based chemotherapy, most patients experience recurrence and disease progression within 2 years, leading to multiple recurrences and the development of platinum-resistant ovarian cancer^2,3^. Therefore, extending the progression-free period and improving the 5 years survival rate are urgent challenges.

Poly (ADP-ribose) polymerase (PARP) inhibitors have emerged as a significant breakthrough in managing advanced ovarian cancer in recent years^4^. PARP is an enzyme crucial for repairing single-strand DNA breaks. PARP inhibitors are a class of drugs that block PARP enzyme activity, causing the accumulation of single-strand breaks, which eventually turn into double-strand breaks (DSBs). DSBs can be repaired by a homologous recombination repair (HRR) pathway. However, in cancer cells with *BRCA* mutations or homologous recombination deficiency (HRD), the HRR pathway is already impaired. Thus, when PARP inhibitors are used to treat these cells, they further compromise DNA repair mechanisms by blocking the repair of single-strand breaks. This creates a state of synthetic lethality, as the combined effect of the impaired HRR pathway and PARP inhibition induces excessive DNA damage, causing selective cancer cell death^5^.

The main adverse effects of PARP inhibitors include hematologic toxicity such as anemia, thrombocytopenia, and neutropenia; gastrointestinal symptoms such as nausea, vomiting, and diarrhea; malaise and fatigue; renal dysfunction; and taste disorder. In addition, increased risk of myelodysplastic syndrome and acute myeloid leukemia is also a characteristic and serious adverse effect caused by long-term use of PARP inhibitors^6^. Based on the results of previous clinical trials and real-world data, many adverse effects of PARP inhibitors are common, most of which appear within the first 3 months of administration. Concerning the characteristics of hematologic toxicity, it is known that anemia is most common with olaparib and thrombocytopenia is most common with niraparib^7^. Regarding the prediction of interruptions due to hematologic toxicity, the dose for niraparib was individualized according to body weight and platelet levels at the time of administration, based on the results of the NOVA study^8^. As for olaparib-induced anemia, there is a report of an association between the daily dose per body weight and the occurrence of anemia^9^. However, no reports on the prediction of adverse effects using the results of blood sampling at the time of the first visit have been identified.

In Japan, olaparib has received approval for various maintenance treatments, including platinum-sensitive relapsed ovarian cancer in 2018^10,11^, *BRCA* mutations following remission of first-line platinum chemotherapy in 2019^12^, and HRD in combination with bevacizumab after remission of first-line platinum chemotherapy in 2020^13^. Conversely, niraparib was approved for maintenance treatment of platinum-sensitive relapsed ovarian cancer in 2020^14^, maintenance treatment following remission of first-line platinum chemotherapy in 2020^15^, and monotherapy treatment of HRD and platinum-sensitive relapsed ovarian cancer after third or more chemotherapy sessions in 2020^16^.

Since eligibility criteria restrict patient enrollment in clinical trials and the adverse effects observed may vary due to racial differences, clinical trial results do not necessarily correspond to real-world practice. Thus, there is growing interest in using real-world data to answer clinical questions unanswerable through clinical trial data^17,18^. Furthermore, accumulating real-world data may reveal findings unavailable in clinical trials or even overturn clinical trial data. Since maintenance therapy follows an initial treatment, accumulating the clinical data takes time. Olaparib and niraparib have been used for 5 and 2 years, respectively, in Japan. There have been several reports of real-world data on the safety of PARP inhibitors for ovarian cancer, and the safety profile for each drug has been clarified^19–24^. Most reports indicate that these drugs are safe to use, with no major differences from adverse effects in previous clinical trials, but they contain little data from Japanese patients, and further accumulation of data is needed. There are populations for whom both of those two drugs can be administered, and it is often difficult to choose among them. In addition, PARP inhibitors share several common adverse effects because of a class effect including nausea, fatigue, and myelotoxicity, but there are differences because of variations in their poly-pharmacology and off-target effects^6^, and it is critical to understand the differences in adverse effects of these drugs to manage the treatment. Therefore, we examined the real-world data on the safety of both drugs for Japanese patients. Additionally, we assessed whether interruptions could be predicted and whether certain trends existed among patients who interrupted the drugs.

## Results

### Patient characteristics

The olaparib and niraparib groups comprised 131 and 50 patients, respectively (Table 1). The median age was 59 (30–80) in the olaparib group and 59 (23–80) in the niraparib group, while the median BMI was 21.2 (14.2–32.8) in the olaparib group and 21.9 (14.3–30.4) in the niraparib group. There were no differences in smoking habits or diabetes between both groups, but alcohol consumption was higher in the niraparib group (*p* = 0.01).

**Table1:** Patient characteristics.

	Olaparib (N = 131)	Niraparib (N = 50)	*p* value
Age	59 (30–80)	59 (23–80)	0.57
BMI	21.2 (14.2–32.8)	21.9 (14.3–30.4)	0.54
Smoking	14 (10.7%)	8 (16.0%)	0.59
Drinking	9 (6.9%)	10 (20.0%)	0.01
DM	9 (6.9%)	5 (10.0%)	0.53
Histologic subtype			
Serous	115 (87.8%)	32 (64.0%)	< 0.01
Endometrioid	9 (6.9%)	6 (12.0%)	
Clear	5 (3.8%)	5 (10.0%)	
Carcinosarcoma	0 (0.0%)	1 (2.0%)	
Unknown	2(1.5%)	6 (12.0%)	
*BRCA* status			
Positive	26 (19.8%)	2 (4.0%)	0.01
Negative	36 (27.5%)	26 (52. 0%)	
Unknown	69 (52.7%)	22 (44. 0%)	
HRD			
Positive	19 (14.5%)	4 (8.0%)	0.32
Negative	2 (1.5%)	12 (24.0%)	
Unknown	110 (83.9%)	33 (66.0%)	

BMI, body mass index; DM, diabetes mellitus; HRD, homologous recombination deficiency.

The proportion of serous carcinoma was significantly higher in the olaparib group (115/131, 87.8%) compared to the niraparib group (32/50, 64.0%) (*p* < 0.01). The proportion of *BRCA*-positive patients was 26/131 (19.8%) in the olaparib group and 2/50 (4.0%) in the niraparib group (*p* = 0.01), while that of HRD-positive patients was 19/131 (14.5%) in the olaparib group and 4/50 (8.0%) in the niraparib group (*p* = 0.32). In addition, the *BRCA* status was unknown for 69/131 (52.7%) patients in the olaparib group and 22/50 (44.0%) patients in the niraparib group. The HRD status was unknown for 110/131 (83.9%) patients in the olaparib group and 33/50 (66.0%) patients in the niraparib group.

### Treatment history

The patients’ treatment history is shown in Table 2. The median observation period was 697 (68–1699) d in the olaparib group and 423 (66–726) d in the niraparib group. The median treatment duration was 190 (14–1667) d in the olaparib group and 203 (5–726) d in the niraparib group. Treatment was discontinued due to adverse effects in 22/131 (16.8%) patients in the olaparib group and 6/50 (12.0%) patients in the niraparib group. The response to the most recent treatment was similar in both groups.

**Table 2:** Treatment history.

	Olaparib (N = 131)	Niraparib (N = 50)	*p* value
Observation days (days)	697 (68–1699)	423 (66–726)	
Duration to the treatment (days)	190 (14–1667)	203 (5–726)	
Reason for termination			
PD	63 (48.1%)	29 (58.0%)	
Adverse effect	22 (16.8%)	6 (12.0%)	
Others	2 (1.5%)	1 (2.0%)	
Number of previous chemotherapy regimens			
1–4	114 (87.0%)	49 (98.0%)	
5–9	13 (9.9%)	1 (2.0%)	
10–	4 (3.1%)	0 (0.0%)	
Response to most recent treatment			
CR	62 (47.3%)	27 (54.0%)	0.51
PR	69 (52.7%)	23 (46.0%)	
Maintenance for first-line chemotherapy	31 (23.7%)	28 (56.0%)	< 0.01
Treatment with Bev	18 (13.7%)	-	
No surgery	5 (3.8%)	10 (20.0%)	< 0.01
Interruption	68 (51.9%)	32 (64.0%)	0.87
Reason for interruption			
Anemia	33 (48.5%)	8 (25.0%)	
Neutropenia	17 (25.0%)	5 (15.6%)	
Thrombocytopenia	6 (8.8%)	16 (50.0%)	
Fatigue	14 (20.6%)	2 (6.3%)	
Nausea	11 (16.2%)	0 (0.0%)	
Others	8 (11.8%)	7 (21.9%)	

CR, complete response; PR, partial response; PD, progressive disease.

Furthermore, 31/131 (23.7%) in the olaparib group and 28/50 (56.0%) in the niraparib group received maintenance therapy following first-line chemotherapy. Maintenance therapy in combination with bevacizumab was administered to 18/131 (13.7%) patients in the olaparib group. Additionally, 5/131 (3.8%) patients in the olaparib group and 10/50 (20.0%) patients in the niraparib group were transferred to maintenance therapy without surgery (*p* < 0.01). Treatment was interrupted in 68/131 (51.9%) patients in the olaparib group and 32/50 (64.0%) patients in the niraparib group (*p* = 0.87). The most common reason for treatment interruption based on blood data was anemia in 33/68 (48.5%) patients in the olaparib group and thrombocytopenia in 16/32 (50.0%) patients in the niraparib group. In the olaparib group, 20/68 (29.4%) patients interrupted treatment due to fatigue and/or nausea.

The characteristics of cases where treatment was discontinued due to adverse effects in the early treatment stage are shown in Table 3. A total of 13/131 (9.9%) patients in the olaparib group and 5/50 (10.0%) patients in the niraparib group discontinued treatment due to adverse effects within the first 3 months of treatment. The median treatment duration was 43 (14–77) days in the olaparib group and 27 (5–28) days in the niraparib group. The median BMI was 20.8 (14.2–29.5) in the olaparib group and 20.5 (14.3–24.9) in the niraparib group, suggesting that both groups had a higher proportion of thin patients compared to the overall population. In both groups, most patients received maintenance therapy after recurrence. In particular, the rate of maintenance therapy after relapse was higher in the niraparib group (80.0%) compared to the overall population (44.0%). There was no difference in the median number of prior chemotherapy regimens in both groups. The most common reason for early treatment discontinuation was fatigue or vomiting in 8/13 (61.5%) patients in the olaparib group and thrombocytopenia in 3/5 (60.0%) patients in the niraparib group. In addition, there were no patients in both groups who withdrew from the drug due to lymphocytopenia.

**Table 3:** The characteristics of patients who discontinued treatment due to adverse effects within the first 3 months of treatment.

	Olaparib (N = 131)	Niraparib (N = 50)
Discontinued cases	13 (9.9%)	5 (10.0%)
Duration to the treatment(days)	43 (14–77)	27 (5–28)
BMI	20.8 (14.2–29.5)	20.5 (14.3–24.9)
Smoking	1 (7.7%)	1 (20.0%)
Drinking	0 (0.0%)	0 (0.0%)
DM	1 (7.7%)	0 (0.0%)
Number of previous chemotherapy regimens	2 (1–6)	2 (1–3)
Response to most recent treatment		
CR	9 (69.2%)	1 (20.0%)
PR	4 (30.8%)	4 (80.0%)
Maintenance for first-line chemotherapy	3 (23.1%)	1 (20.0%)
Reason for termination		
Anemia	2 (15.4%)	0 (0.0%)
Neutropenia	1 (7.7%)	0 (0.0%)
Thrombocytopenia	0 (0.0%)	3 (60.0%)
Fatigue, Vomiting	8 (61.5%)	1 (20.0%)
Dysgeusia	1 (7.7%)	1 (20.0%)
Interstitial pneumonia	1 (7.7%)	0 (0.0%)

BMI, body mass index; DM, diabetes mellitus; CR, complete response; PR, partial response.

### Hematological data trends

**Figure 1:**
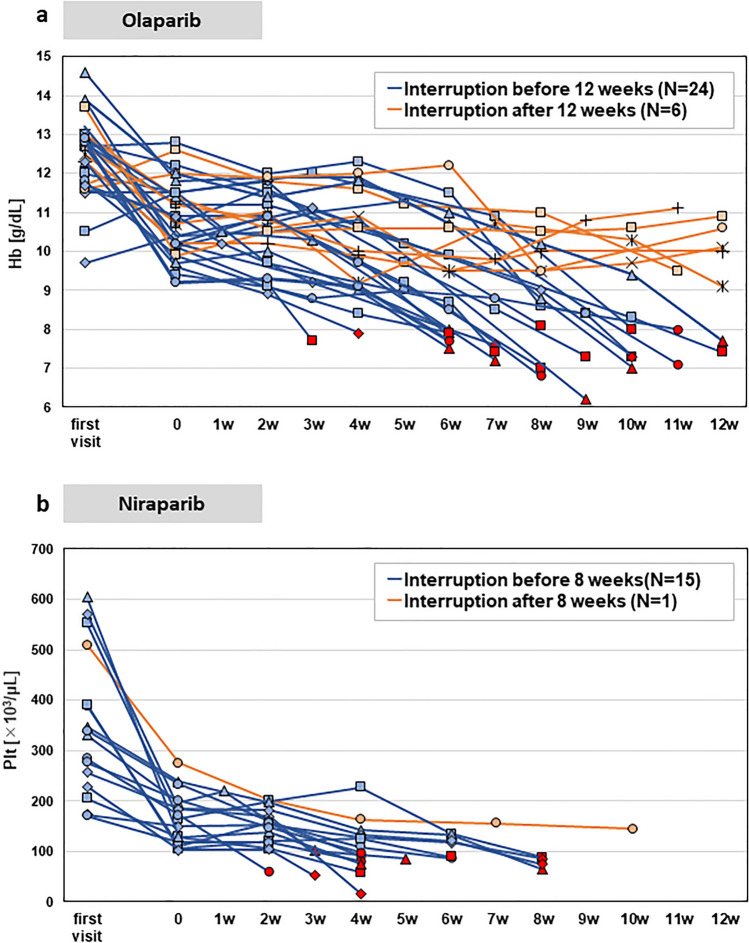
(**a**) The trends of blood hemoglobin levels in the olaparib group are shown. There were 30 cases of initial interruption of treatment due to anemia after the start of treatment. Treatment was interrupted when the hemoglobin level fell below 8 mg/dL. A total of 22/30 (73.3%) patients was interrupted within 4–12 weeks of treatment. (**b**) The trends of blood platelet levels in the niraparib group are shown. There were 16 cases of initial interruption of treatment due to thrombocytopenia after the start of treatment. Treatment was interrupted when the platelet level fell below 100,000/µL. A total of 15/16 (73.3%) patients was interrupted within 8 weeks of treatment.

The trends of blood hemoglobin and platelet levels, which were the most common causes of treatment interruption based on blood data, in the olaparib and niraparib groups, respectively, are shown in Fig. 1a. In the olaparib group, there were 30 cases of initial treatment interruption due to anemia after the start of treatment. Among these, 22/30 (73.3%) were interrupted within 4–12 weeks of treatment. Conversely, there were 16 cases of initial treatment interruption due to thrombocytopenia in the niraparib group (Fig. 1b). Among these, 15/16 (93.8%) were interrupted within 8 weeks of treatment. Interruptions due to thrombocytopenia in the niraparib group tended to occur earlier than interruptions due to anemia in the olaparib group. As described above, the timing of interruptions showed certain trends and characteristics for each drug.

### Prediction of interruption

**Figure 2:**
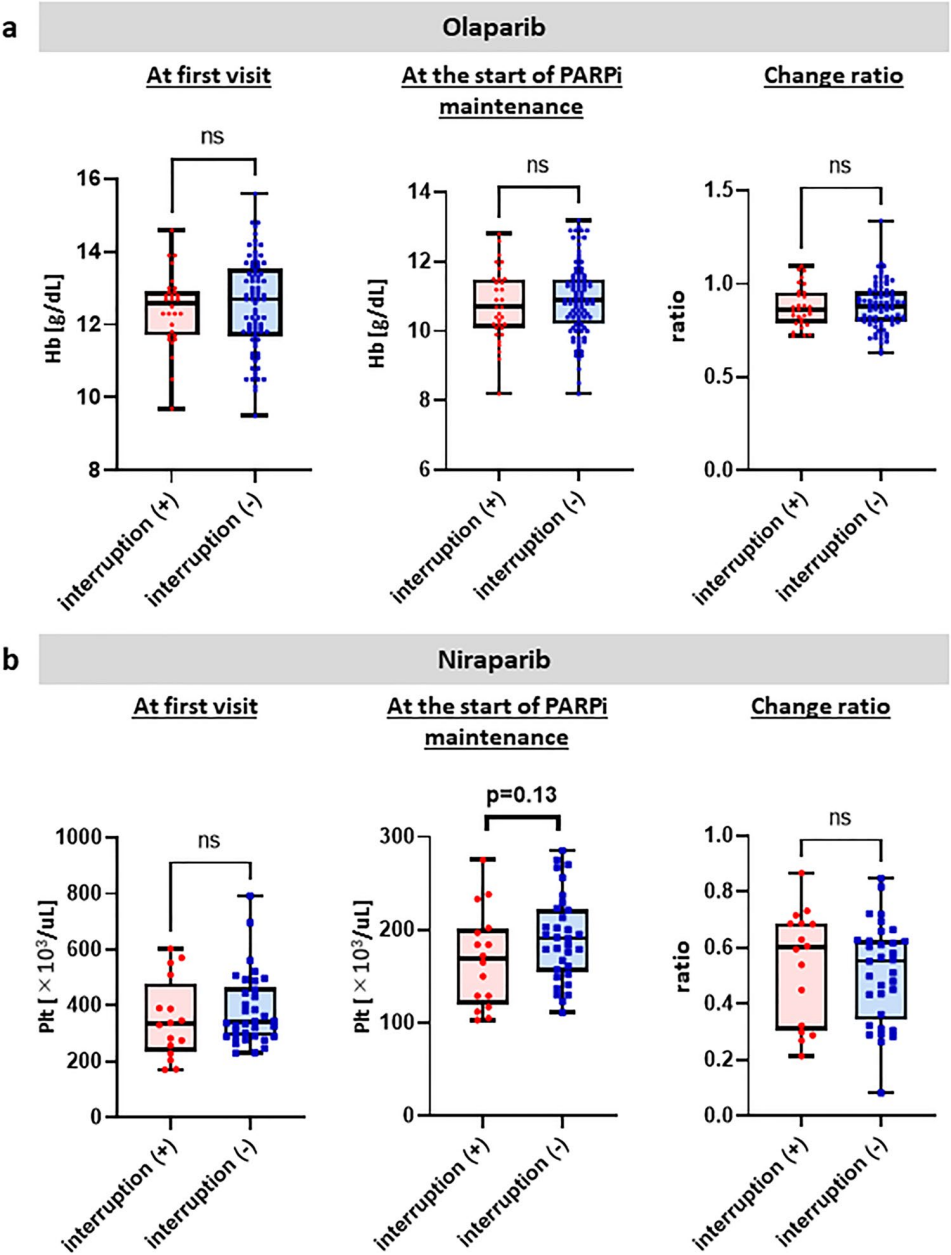
(**a**) Hemoglobin values in the olaparib group at the initial visit and at the start of treatment, and ratio of change of the two points are shown with and without interruption. (**b**) Platelet values in the niraparib group at the initial visit and at the start of treatment, and ratio of change of the two points are shown with and without interruption.

Predicting adverse effects in patients would enhance treatment management and ensure safe administration of the drug, leading to successful completion of the treatment. We hypothesized that the degree of adverse effects during chemotherapy might correlate with that during maintenance therapy using consecutive PARP inhibitors. Therefore, to predict treatment interruption due to hematological adverse effcts, blood collection data before treatment and after chemotherapy were investigated. Specifically, we compared the blood collection data at the initial visit and at the start of olaparib/niraparib treatment and the rate of change between the groups with and without interruption. The results for interruption due to anemia in the olaparib group are shown in Fig. 2a. No significant differences existed between both groups, and predicting interruption due to anemia from the blood data at the initial visit or the start of treatment was challenging. The results for interruption due to thrombocytopenia in the niraparib group are shown in Fig. 2b. No significant differences existed between both groups, and predicting interruption due to thrombocytopenia from the blood data at the initial visit or the start of treatment was challenging. Both olaparib-induced interruption due to anemia and niraparib-induced interruption due to thrombocytopenia were difficult to predict from the blood data.

## Discussion

Due to the high recurrence rate and low survival rate of ovarian cancer, as well as the limited availability of drugs other than chemotherapy in the past, the emergence of PARP inhibitors has brought hope for ovarian cancer treatment^25^. We aimed to explore the real-world data of olaparib and niraparib in Japan, as both agents target the same pathways but are used differently. Olaparib is primarily used as a single agent in maintenance therapy after first-line chemotherapy for *BRCA*-positive patients, based on the SOLO-1 study^12^, and in combination with bevacizumab for HRD patients, based on the PAOLA-1 study^13^. Conversely, niraparib can be used regardless of biomarkers, based on the PRIMA study^15^. In this study, the rate of serous carcinoma was considerably lower than in the clinical trial^15^, possibly since niraparib is covered by insurance regardless of *BRCA* or HRD status. Since the approval of the PAOLA regimen in 2020, patients with HRD have essentially used olaparib, so it was thought that there would be fewer serous, *BRCA*-positive status, and HRD patients in the niraparib group in this study because *BRCA* mutations and HRD are frequently observed in high-grade serous ovarian carcinoma^26^. For the same reason, it is likely that more patients in the niraparib group who could not undergo surgery and whose tissue samples could not be obtained received niraparib therapy. The complete response rate after the most recent platinum-based chemotherapy was 47.3% in the olaparib group and 54.0% in the niraparib group. When limited to patients receiving maintenance therapy after first-line chemotherapy, the rate was 93.5% in the olaparib group and 60.7% in the niraparib group, similar to prior studies^12,13,15^.

In this study, there were differences in adverse effects between the olaparib group and the niraparib group. Despite sharing the same pharmacological mechanism, the toxicity profile is different for both agents^27,28^. The differences in adverse effects of these agents could be attributed to dosage schedule, half-life, drug interactions, and metabolism^6^. In this study, Grade 3 or 4 adverse reactions in the olaparib group included anemia (25.2%), neutropenia (14.5%), thrombocytopenia (3.8%), and fatigue/nausea (15.3%), occurring more frequently than in previous studies^10–13^. In particular, the findings were notable for the high number of dose interruptions due to fatigue and nausea. Nausea and fatigue were the cause of 61% of patients who discontinued the drug within 3 months of the start of administration, which is considerably higher than in previous clinical trials and real-world data^7,24^. Similarly, 8.4% of all cases were terminated due to nausea and fatigue, which is also higher than previously reported^24^. These results were more frequent than the data from China, which is also Asian^19^. This discrepancy might be because the Japanese have a lower BMI than Westerners, which might be caused by their food habits. It has been reported that energy balance including body composition and nutritional status could influence on pharmacokinetics of cancer therapeutics^29^, and the habits may influence racial differences in these adverse effects. Nausea and fatigue can affect quality of life particularly if persistent, and it is therefore important to educate and inform the patient of these adverse effects, and proactive efforts should be taken to prevent and treat nausea. Conversely, Grade 3 or 4 adverse reactions in the niraparib group comprised anemia (14.0%), neutropenia (10.0%), thrombocytopenia (32.0%), and fatigue/nausea (6.0%), respectively, with these results either being the same or less frequent than in previous studies^14,15,30^. This may be because the starting dose for niraparib was individualized based on body weight and platelet count. Previous studies started with a fixed dose of 300 mg, and the NOVA trial results led to the individualization of the initial dose according to body weight and platelet count. Moreover, recently published data from the NORA trial confirmed that dose individualization is associated with improved hematologic toxicity^31^.

Thrombocytopenia is a peculiar toxicity observed in niraparib treatment, and we showed that niraparib-induced thrombocytopenia occurs earlier than olaparib-induced anemia. Furthermore, there were three cases where platelet levels did not recover after treatment interruption, leading to the discontinuation of the drug. This finding suggests that niraparib-induced thrombocytopenia may be more robust than olaparib-induced anemia. Among patients who discontinued treatment due to adverse effects, 59% in the olaparib group and 100% in the niraparib group discontinued within the first 3 months of treatment. It was considered that thin patients with several previous regimens should be managed with particular attention to adverse effects early in the treatment. Furthermore, careful long-term management of adverse effects is crucial, especially when treated with olaparib.

While there have been many reports on the adverse effects of PARP inhibitor therapy^32,33^, no reports describing their predictability using blood sampling data at the initial visit exist. This is the first study on the predictability of interruptions in PARP inhibitor therapy using blood collection data both at the initial visit and at the start of administration. However, the blood toxicity of both agents was difficult to predict using these data. Generally, hematological adverse events associated with PARP inhibitors are frequent but transient, occurring during the first months of therapy, and are often resolved with dose reduction. We also observed hematological adverse events in the later treatment stages, especially in the olaparib group, and rare complications such as myelodysplastic syndrome and acute myeloid leukemia^34^. Thus, regular blood tests should be conducted even after the initial months of treatment.

In conclusion, we examined the real-world data on the safety of olaparib and niraparib, as well as the predictability of intake interruption based on blood sampling data. The novelty of this paper is that it presents and characterizes a large real-world data set of Japanese patients and that it examines the possibility of treatment interruption using blood sampling data at the initial visit. Patient backgrounds and toxicity profiles differed between the olaparib and niraparib groups. In particular, the current results were characterized by increased adverse events in olaparib. However, predicting the blood toxicity of both agents using blood collection data was challenging and the findings suggested that patients with low BMI and patients on maintenance therapy after relapse may require particular attention to the occurrence of adverse effects. This study revealed the characteristics of the patients and the timing of interruption for each drug and highlighted the importance of carefully managing adverse effects, particularly during the early treatment stages.

### Patients and methods

The records of 181 patients with ovarian cancer who received olaparib and/or niraparib treatment at Nagoya University Hospital (Nagoya, Japan) and Aichi Cancer Center Hospital (Nagoya, Japan) from May 2018 to December 2022 were retrospectively reviewed. Both the olaparib and niraparib groups included patients undergoing maintenance treatment for advanced epithelial ovarian, fallopian tube, or primary peritoneal cancer after first-line platinum-based chemotherapy and recurrent epithelial ovarian, fallopian tube, or primary peritoneal cancer after platinum-based chemotherapy. We investigated the patients’ clinical information, including age, body mass index (BMI), smoking and drinking habits, histological type, *BRCA* and HRD status, previous chemotherapy regimens, adverse effects, and blood sampling data, at several points. Germline *BRCA1/2* status was assessed using BRACAnalysis CDx^®^ (Myriad Genetics, Inc., Salt Lake City, UT, USA). HRD status was determined using MyChoice^®^ CDx (Myriad Genetics, Inc., Salt Lake City, UT, USA). The criteria for drug withdrawal were following the respective guides for proper use and withdrawals were made at the onset of grade 3 or 4 adverse effects, except for interstitial pneumonia for olaparib and thrombocytopenia for niraparib. This study was approved by the Ethics Committee of Nagoya University and the Ethics Committee of Aichi Cancer Center (Approval no. 2013-0078). All methods were performed in accordance with the relevant guidelines and regulations as well as in compliance with the requirements of the Declaration of Helsinki. Furthermore, informed consent was obtained from all participants.

Statistical analyses were performed using GraphPad Prism 9, with the Mann–Whitney U test or chi-square test used for comparisons between both groups.* p* < 0.05 was considered statistically significant.

## Data Availability

All data generated or analyzed during this study are included in this published article.
